# Associations between night/shift working and late-life brain health

**DOI:** 10.1093/braincomms/fcaf264

**Published:** 2025-07-04

**Authors:** Josh King-Robson, Jennifer M Nicholas, Sarah-Naomi James, Ashvini Keshavan, Dylan M Williams, James Groves, Carole H Sudre, Kirsty Lu, Josephine Barnes, William Coath, David M Cash, Sarah E Keuss, Marcus Richards, Jason D Warren, Jonathan M Schott

**Affiliations:** Dementia Research Centre, UCL Queen Square Institute of Neurology, University College London, London, WC1N 3BG, UK; Department of Medical Statistics, London School of Hygiene & Tropical Medicine, University of London, London WC1E 7HT, UK; Dementia Research Centre, UCL Queen Square Institute of Neurology, University College London, London, WC1N 3BG, UK; MRC Unit for Lifelong Health and Ageing at UCL, University College London, London WC1E 7HB, UK; Dementia Research Centre, UCL Queen Square Institute of Neurology, University College London, London, WC1N 3BG, UK; MRC Unit for Lifelong Health and Ageing at UCL, University College London, London WC1E 7HB, UK; Dementia Research Centre, UCL Queen Square Institute of Neurology, University College London, London, WC1N 3BG, UK; Dementia Research Centre, UCL Queen Square Institute of Neurology, University College London, London, WC1N 3BG, UK; Dementia Research Centre, UCL Queen Square Institute of Neurology, University College London, London, WC1N 3BG, UK; Dementia Research Centre, UCL Queen Square Institute of Neurology, University College London, London, WC1N 3BG, UK; Dementia Research Centre, UCL Queen Square Institute of Neurology, University College London, London, WC1N 3BG, UK; Dementia Research Centre, UCL Queen Square Institute of Neurology, University College London, London, WC1N 3BG, UK; Dementia Research Centre, UCL Queen Square Institute of Neurology, University College London, London, WC1N 3BG, UK; MRC Unit for Lifelong Health and Ageing at UCL, University College London, London WC1E 7HB, UK; Dementia Research Centre, UCL Queen Square Institute of Neurology, University College London, London, WC1N 3BG, UK; Dementia Research Centre, UCL Queen Square Institute of Neurology, University College London, London, WC1N 3BG, UK; MRC Unit for Lifelong Health and Ageing at UCL, University College London, London WC1E 7HB, UK; UK Dementia Research Institute at UCL, University College London, London NW1 3BT, UK

**Keywords:** Alzheimer’s disease, sleep, circadian rhythms, shift work, dementia

## Abstract

Sleep and circadian disturbances are associated with increased dementia risk. The mechanism remains poorly understood. We aimed to examine the relationship between night/shift working at age 31 and biomarkers of late-life brain health and to estimate the extent to which these relationships are mediated by unhealthy lifestyle behaviours. A prospective longitudinal cohort study, Insight 46, recruited participants from the Medical Research Council National Survey of Health and Development (NSHD) 1946 British Birth cohort. All born in the same week in 1946, participants were assessed at age 70 with multi-modal structural and molecular brain imaging and fluid biomarkers, from which whole-brain and hippocampal volumes, white matter hyper-intensity volume (WMHV), ^18^F-florbetapir amyloid-β PET Centiloids and plasma phosphorylated tau (p-tau)217 were derived. Prospective data collection included night/shift working at age 31, alongside smoking, alcohol intake, body mass index, exercise, blood pressure, Framingham risk score (FRS) at multiple timepoints from age 20 to 70 and dementia diagnosis or death by age 78. Analyses were adjusted for sex, age, education, socioeconomic position and, where appropriate, total intracranial volume or apolipoprotein E (*APOE*) genotype. Night/shift working data were available for 431 Insight 46 participants {50% female, mean age 70.7 years [standard deviation (SD) 0.6]}. Night/shift workers had lower whole-brain volume [−19.9 mL, 95% confidence interval (CI) −31.9, −7.9, *P =* 0.001], lower amyloid PET Centiloids (−9.45, 95% CI −14.7, −4.1, *P =* 0.0008) and lower plasma p-tau217 concentration (−0.05 pg/mL, 95% CI −0.10, −0.001, *P =* 0.04), without significant difference in hippocampal volume or WMHV. p-tau217 concentrations were also lower in night/shift workers from a wider sample from the NSHD cohort [*n* = 1067, mean age 69.9 (SD 0.7), −0.05 pg/mL, 95% CI −0.08, −0.02, *P =* 0.004]. By age 78, night/shift workers in the NSHD cohort (*n* = 3040) had lower rates of all-cause (excluding vascular) dementia (hazard ratio 0.33, 95% CI 0.12, 0.92, *P* = 0.03). Night/shift workers had 0.6% higher FRS (*P =* 0.01) at age 36, smoked 5.9 more pack-years by age 53 (*P =* 0.005), consumed 10.7 g/day more alcohol by age 63 (*P =* 0.006) and had higher rates of *APOE* ɛ4 allele carriage. Lifestyle behaviours mediated 28% of the lower brain volume in night/shift workers. Despite less healthy lifestyles, higher rates of *APOE* ɛ4 allele carriage and smaller brains, night/shift workers had lower levels of Alzheimer’s disease pathology as measured by amyloid PET and plasma p-tau217 and approximately one-third of the risk of dementia by age 78 compared with non-night/shift workers. Lower brain volume in night/shift workers was partially mediated by unhealthy behaviours. Reduced dementia risk in night/shift workers is unexpected and will require further study.

## Teaser

King-Robson *et al*. find that despite having less healthy lifestyles, higher rates of *APOE* ɛ4 allele carriage and smaller brains, night/shift workers had lower levels of Alzheimer’s disease pathology as measured by amyloid PET and plasma p-tau217 at age 70 and one-third the risk of dementia by age 78.

## Graphical Abstract

**Graphical Abstract:**
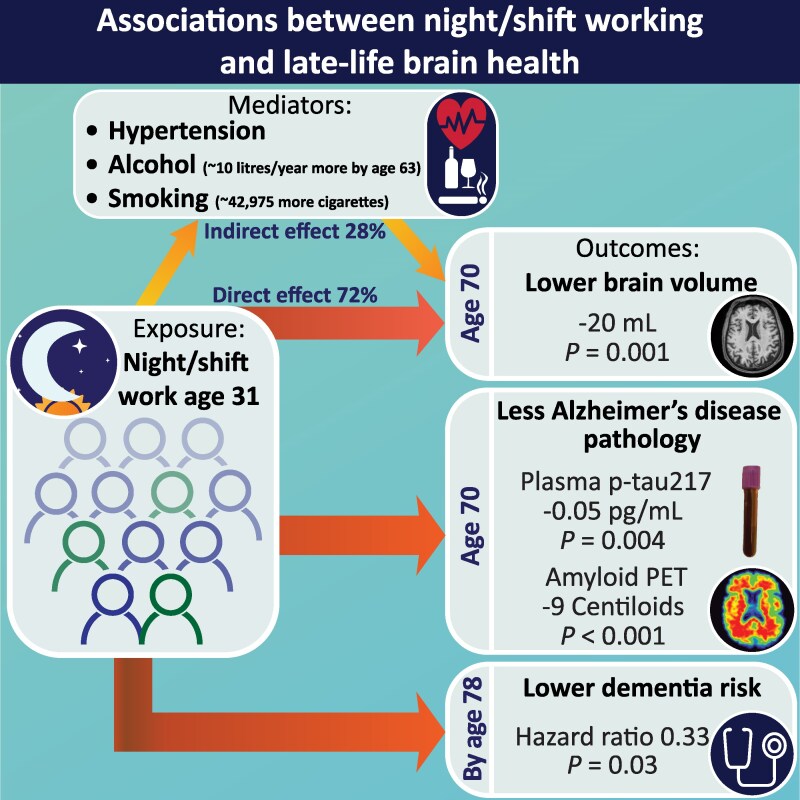

## Introduction

There is growing evidence for a role of sleep and circadian disruption in neurodegenerative disease, particularly Alzheimer’s disease.^1-4^ Shift workers experience regular sleep and circadian disruption but also have increased rates of adverse lifestyle behaviours, making them an ideal group in which to examine relationships between sleep and circadian disruption, health behaviours, neurodegeneration and dementia risk.

Studies demonstrating abnormal levels of amyloid-β (Aβ) in cerebrospinal fluid and with PET after acute sleep deprivation have suggested the increased Alzheimer’s disease risk in those with sleep disturbance may be mediated by amyloidogenic pathways.^5,6^ However, it remains unclear whether acute biochemical changes observed in experimental paradigms equate to long-term amyloid accumulation in humans under physiological conditions, while the chronic effects of circadian disruption are poorly understood. Other mechanisms may also be important; sleep and circadian disruption impair learning and memory,^7,8^ increase markers of neuronal damage^9-11^ and are associated with neuroendocrine and immune dysregulation, oxidative stress and alterations in cellular metabolism.^12-16^

Shift workers provide an opportunity to study the effects of chronic sleep and circadian disruption in a natural setting. Studies reporting increased dementia risk among shift workers have raised the prospect that sleep and circadian disruption may play a role in the neurodegenerative process.^1,17-19^ Shift working is also associated with a range of unhealthy lifestyle behaviours, themselves associated with dementia risk. It remains unclear to what extent dementia risk in shift workers is secondary to increased rates of these unhealthy behaviours or due to the chronic effects of sleep and circadian disruption directly.^20-24^

In this study, we combined prospectively collected life course data, including night/shift working age 31, with multi-modal structural and molecular brain imaging and fluid biomarkers collected ∼40 years later, in a cohort of individuals all born in the same week in 1946. We examined the relationship and potential causal pathways linking sleep disturbance in early mid-life with brain health and dementia risk in late-life.

## Materials and methods

**Figure 1:**
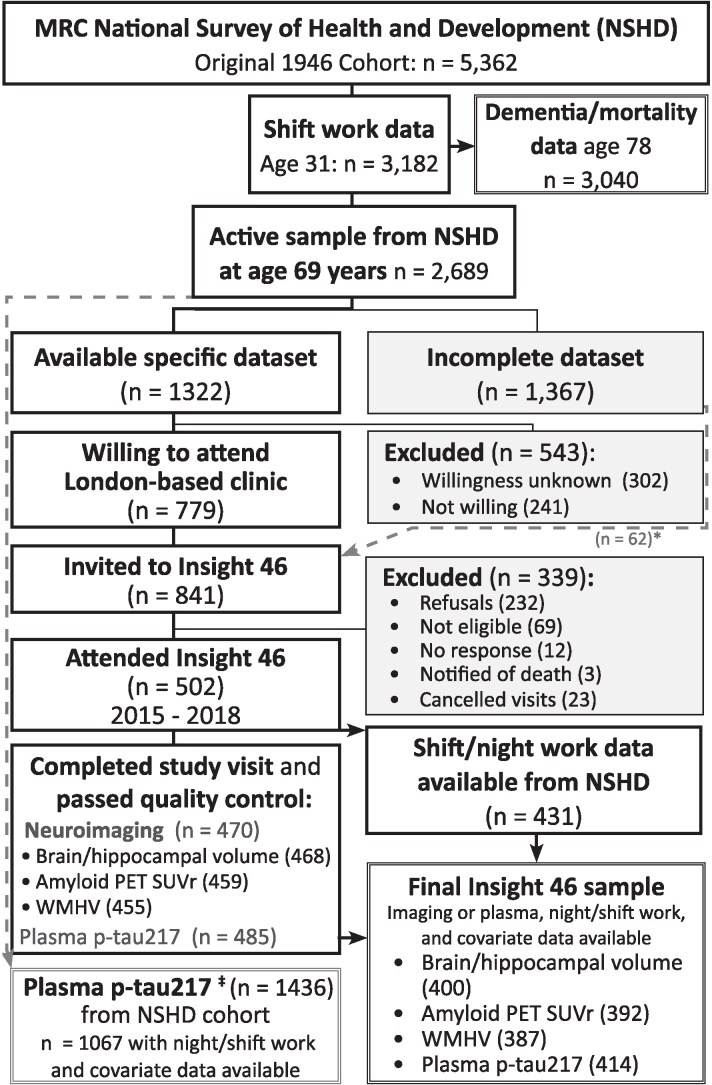
**Flowchart of recruitment and data acquisition.** NSHD participants were invited to participate in Insight 46 where a minimum dataset of life course data was available; this was subsequently relaxed to include those *n* = 62* where a previous measure of lung function, smoking or physical exercise was unavailable to meet the target sample size.^27^ Plasma p-tau217^‡^ from participants attending Insight 46 (*n* = 485) was supplemented by samples taken from the wider NSHD cohort (*n* = 951) during the same collection period.

The Medical Research Council (MRC) National Survey of Health and Development (NSHD) is the longest continuously running birth cohort in the world, initially comprising 5362 individuals, all born in mainland Britain in the same week in 1946. By age 69, participants had completed 24 waves of prospective data collection.^25^ The Insight 46 neuroimaging sub-study recruited 502 NSHD participants at age ∼70 to undergo multi-modal neuroscience assessment (Fig. 1).^26,27^

### Protocol approvals, registrations and patient consents

This research has approval from the National Research Ethics Service Committee London (REC reference 14/LO/1173). All participants provide written informed consent.

### Sleep/circadian variables

At age 31, NSHD participants were asked if they were currently undertaking night or shift work (yes/no) and how many nights per month they work after midnight. Night workers (as opposed to night/shift workers) were identified where participants reported working ≥1 night per month after midnight.

Self-reported sleep quality was assessed in the NSHD at ages 31, 36, 43, and 69. At age 31, participants were asked if in the last month they have had any trouble with sleep (yes/no), and at age 36, participants were asked if they had trouble initiating sleep in the last month (no/≥1 h/≥2 h delay), and those who reported sleep disturbance or depression were asked if they wake early (no/≥1 h/≥2 h). Greater than 1 and ≥2 h are combined in all analyses to represent disturbed sleep. At age 43, participants were asked if they have trouble getting to sleep and if they have trouble with waking up and not being able to get back to sleep (never/occasionally/sometimes/quite often/very often/always). At age 69, sleep quality was assessed using the Pittsburgh Sleep Quality Index (PSQI).^28^

At age 73, Insight 46 participants were invited to undergo 7 nights of wrist actigraphy (Actiwatch Spectrum Plus, Philips). Data were collected in 30-s epochs, and the first night was excluded due to ‘first night’ effects, from which total sleep time (TST, hours), sleep efficiency (SE, %), wakefulness after sleep onset (WASO, minutes), interdaily stability (IS) and intradaily variability (IV) were calculated.

### Covariables

Educational attainment was determined as the highest qualification achieved by age 26, grouped into five categories.^29^

Adult socioeconomic position was determined at age 53, grouped as per Office of Population Censuses and Surveys classification into six categories.

Childhood cognition was measured at age 8 combining four tests of verbal/non-verbal ability, converted into a *Z*-score based on the full NSHD cohort.^29^ Where unavailable, equivalent scores at age 11 or 15 were used.

Apolipoprotein E *(APOE)* ɛ4 carrier status was classified as ε4 carrier or non-ε4 carrier.^26^

### Potential health mediators

Blood pressure was measured at ages 36, 43, 53, 63 and 69.^30^ Hypertension was defined as an average of two readings >140/90 mmHg or anti-hypertensive medication use.

Smoking habits were self-reported at ages 20, 25, 31, 36, 43, 53 and 63, from which pack-years were calculated.^31^

Body mass index (BMI) was calculated from height and weight measured at ages 36, 43, 53, 63 and 70.

Leisure time physical activity was self-reported at ages 36, 43, 53, 63 and 69, categorized as inactive, less active (1–4 episodes/month) or more active (>4 episodes/month).^32^

Office-based Framingham Heart Study–cardiovascular risk scores (FRS) were calculated at ages 36, 53 and 70, estimating a 10-year risk of cardiovascular events. The FRS combines age, blood pressure, anti-hypertensive treatment, smoking and diabetes status, without serum cholesterol which was unavailable at age 36.^33^

Alcohol consumption (grams/day) was calculated from self-reported 7-day food diaries at ages 36, 43, 53 and 63.^32^

### Imaging variables

All imaging was performed on the same Biograph mMR 3T PET/MRI scanner (Siemens Healthcare), enabling simultaneous acquisition of dynamic Aβ PET and MR data over a single 60-min scanning session following injection of 370 MBq of ^18^F-florbetapir.^26^

Imaging analysis utilized automated pipelines with manual quality control and correction. Briefly, whole-brain and hippocampal volumes were calculated from volumetric T1 data (1.1-mm isotropic) using brain Multi-Atlas Propagation and Segmentation and Similarity and Truth Estimation for Propagated Segmentations.^26,34,35^ Total intracranial volume (TIV) was calculated with statistical parametric mapping.^36^ Global white matter hyper-intensity volume (WMHV) was calculated from volumetric T1 and fluid-attenuated inversion recovery images using Bayesian Model Selection.^30,37^

^18^F-Florbetapir Aβ PET standardized uptake volume ratio (SUVr) was calculated using predefined composite neocortical target and whole-cerebellum reference regions following partial volume correction using the iterative Yang method and converted to Centiloid scale.^30,38-40^ Aβ status was determined from SUVr data with a Gaussian mixture model approach, using the 99th percentile of the lower Gaussian as the cut-point.

### Plasma phosphorylated tau 217

Blood was taken from Insight 46 participants at age ∼70 and other NSHD participants during a home visit at age 69.^26^ Samples from age 69 were selected for assaying to prioritize those who subsequently passed away, to enable measurement of phosphorylated tau at threonine 217 (p-tau217) concentrations in individuals who have no prospect of joining Insight 46 (where further p-tau217 assaying is planned). Levels of p-tau217 were determined in both sets of plasma samples in singlicate using the ALZpath Simoa V2 assay (Quanterix),^41^ and values were adjusted for cross-run variation within each sample set using pooled plasma control samples assayed on each run. As part of the assaying of p-tau217 on samples from age 69, 18 plasma samples previously assayed in singlicate in Insight 46 and 2 pooled plasma samples (also used in the previous Insight 46 assaying) were re-assayed in duplicate to allow for bridging of the NSHD data to the Insight 46 results.

### Cognitive and clinical outcomes

Cognition was assessed in Insight 46 at age ∼70 using the Pre-clinical Alzheimer Cognitive Composite (PACC), which combines the Mini Mental State Examination, Logical Memory IIa from the Wechsler Memory Scale-Revised, Digit-Symbol Substitution test from the Wechsler Adult Intelligence Scale-Revised and 12-item Face-Name test into a score sensitive to pre-clinical cognitive decline.^29^

Date at dementia diagnoses were collected from Hospital Episode Statistics (HES). These nationally collated data include diagnoses at all National Health Service (NHS) funded hospital admissions and outpatient appointments in the UK until age 78.0 years.^42^ Noting that correlation between clinical and pathological diagnosis of neurodegenerative dementias is poor, even in expert centres,^43^ we examined all coded dementia diagnoses together, excluding vascular dementia (International classification of diseases codes F00, F02, F03, G30, G31) and separately all vascular dementia diagnoses (F01).^44^

Mortality data were collected from the NHS central register.^45^

### Statistical analysis

All analysis was performed in R (version 4.3.1).^46^

Descriptive statistics compared participant characteristics in those who did and did not report night/shift working using Welch’s two-sample *t*-test or Wilcoxon rank-sum test (normally/non-normally distributed continuous outcomes) and Fisher’s exact test (categorical outcomes).

To examine whether night/shift working at age 31 was predictive of brain health at age 70, linear regression was conducted for whole-brain, hippocampal and WMHV, Aβ PET Centiloids, plasma p-tau217, PACC and sleep/circadian measures. Where outcomes were non-normally distributed, percentile confidence intervals (CIs) were calculated using case bootstrapping (200 000 replications) with *P*-values inferred from the CIs.^47,48^

All analyses were adjusted for age at imaging/plasma/actigraphy, sex, socioeconomic position and educational attainment, alongside TIV for volumetric measures, *APOE* ɛ4 carrier status for Aβ PET and plasma p-tau217, and childhood cognition for PACC. For interpretability, results are presented as unit-change in biomarker or life course factor in night/shift workers compared with non-shift workers for continuous outcome variables, coefficients are exponentiated to present odds or hazard ratios for binary outcomes. Sensitivity analyses examined significant regression results in those who specifically reported night working, the effects of childhood cognition, *APOE* ɛ4 carrier status, excluded those with significant brain disorders,^26,49^ and examined for an interaction between shift working and sex. Values of *P* < 0.05 were considered statistically significant for all analyses.

Cox proportional cause-specific hazards regression models were used to compare rates of dementia diagnosis in night/shift workers and non-shift workers, censoring for the competing event of death and for age at emigration by age 78.0 years (date of last HES data collection). The timescale used for the analysis was age. The proportional hazards assumptions were confirmed using Schoenfeld residual tests. The g-formula approach in the adjustedCurves package^50^ was used to calculate the cumulative incidence of dementia with and without night/shift working, while accounting for the competing risk of death and adjusting for sex, education and adult socioeconomic position.

### Mediation

To identify candidate mediators of the relationship between night/shift working and brain health, linear regression (continuous risk factors), logistic regression (binary risk factors) and ordered logistic regression (ordinal risk factors) were conducted to examine whether night/shift working was associated with differences in cardiovascular risk factors and alcohol consumption across the life course. Linear regression then examined associations between these same risk factors and imaging metrics that demonstrated associations with night/shift work.

Causal mediation analysis (Medflex)^51^ quantified the extent to which significant effects of night/shift working on imaging outcomes were mediated by selected life course factors. This natural-effects model approach splits the total effect of night/shift working on imaging outcomes into two components: a direct effect of night/shift working in absence of any impact of shift work on cardiovascular risk factors and an indirect effect of night/shift working mediated by its impact on those factors.^51,52^ A parsimonious model selected mediators with the strongest relationship to both shift working and imaging outcomes (*P* ≤ 0.15 within 5 years of night/shift working, *P* ≤ 0.1 at later timepoints) and allowed for interactions between mediators measured at the same timepoint.

Mediation analyses were performed on multiply imputed datasets to mitigate potential bias from missing data, using multiple imputation of covariates by substantive model-compatible fully conditional specification (SMC-FCS, 150 iterations, 150 imputations).^53^ This imputation approach imputes the missing data to be compatible with the specified model for the imaging outcome and so provides consistent results in situations where traditional multiple imputation approaches may produce biased estimates, such as with the interactions we included at each timepoint. The multiple imputation model included all listed covariables, health mediators, total brain volume, TIV, WMHV and Aβ PET Centiloids, imputed within SMC-FCS using linear regression, logistic regression or proportional odds regression, for continuous, binary and ordinal data, respectively.

## Results

### Participant characteristics

Four hundred thirty-one Insight 46 participants had night/shift working data available at age 31, of whom 74 (17%) were night/shift workers. Participant characteristics are summarized in Table 1. There was a significantly higher proportion of males and *APOE* ɛ4 carriers in the night/shift working group but no significant difference in socioeconomic position, educational attainment, childhood cognition or late-life self-reported sleep quality.

**Table 1:** Participant characteristics

	Overall, *N* = 431	Night/shift worker?		
		No, *N* = 357	Yes, *N* = 74^a^	*P*-value
**Characteristics**				
Age at imaging, years, mean (SD)	70.7 (0.6)	70.7 (0.7)	70.6 (0.7)	0.2^b^
Female sex, *n* (%)	214 (50%)	194 (54%)	20 (27%)	<0.001
Adult socioeconomic position, *n* (%)				0.2
Professional	45 (10%)	36 (10%)	9 (12%)	
Intermediate	229 (53%)	187 (52%)	42 (57%)	
Skilled (non-manual)	95 (22%)	85 (24%)	10 (14%)	
Skilled (manual)	38 (8.8%)	28 (7.8%)	10 (14%)	
Partly/unskilled	24 (5.6%)	21 (5.9%)	3 (4.1%)	
Highest educational qualification, *n* (%)				0.8
None	62 (14%)	50 (14%)	12 (16%)	
Below O-levels (vocational)	23 (5.3%)	20 (5.6%)	3 (4.1%)	
O-levels or equivalent (secondary school)	115 (27%)	96 (27%)	19 (26%)	
A-levels or equivalent (higher secondary/college)	155 (36%)	131 (37%)	24 (32%)	
Degree or equivalent	76 (18%)	60 (17%)	16 (22%)	
Childhood cognition, *Z*-score, mean (SD)	0.38 (0.74)	0.39 (0.74)	0.31 (0.72)	0.4^c^
*APOE* ɛ4 carrier, *n* (%)	132 (31%)	101 (28%)	31 (42%)	0.027
PSQI, global score age 69, mean (SD)	4.6 (2.9)	4.7 (2.9)	4.3 (2.5)	0.4^b^
**Life course mediators**				
FRS, % 10-year risk, mean (SD)				
Age 36	2.9 (1.8)	2.7 (1.5)	3.9 (2.4)	
Age 53	12.0 (7.4)	11.4 (6.6)	15.1 (9.8)	
Age 70	28.8 (14.7)	27.8 (14.3)	33.6 (15.6)	
Hypertension, *n* (%)				
Age 36	65 (16%)	48 (14%)	17 (24%)	
Age 43	74 (18%)	54 (16%)	20 (27%)	
Age 53	170 (41%)	134 (40%)	36 (50%)	
Age 63	236 (55%)	193 (54%)	43 (58%)	
Age 69	252 (60%)	207 (59%)	45 (62%)	
Alcohol, g/day, mean (SD)				
Age 36	15 (21)	14 (17)	24 (33)	
Age 43	15 (20)	14 (19)	20 (23)	
Age 53	17 (21)	16 (20)	24 (25)	
Age 63	33 (22)	31 (20)	45 (29)	
Smoking, lifetime pack-years, mean (SD)				
Age 20	0.91 (1.7)	0.84 (1.6)	1.26 (2.3)	
Age 36	4.9 (6.9)	4.3 (6.1)	7.9 (9.5)	
Age 43	6.2 (8.9)	5.4 (7.9)	10.4 (12.5)	
Age 53	7.6 (11.7)	6.5 (10.2)	13.2 (16.5)	
Age 63	8.0 (13.1)	7.0 (11.6)	13.6 (18.4)	
BMI, kg/m^2^, mean (SD)				
Age 36	23.8 (3.2)	23.8 (3.2)	23.8 (3.3)	
Age 43	25.0 (3.4)	25.1 (3.4)	24.9 (3.5)	
Age 53	27.1 (4.3)	27.2 (4.3)	26.9 (3.9)	
Age 63	27.8 (4.5)	27.8 (4.5)	27.8 (4.6)	
Age 70	27.9 (4.7)	27.8 (4.7)	28.0 (4.6)	
Physically inactive, *n* (%)				
Age 36	130 (30%)	110 (31%)	20 (27%)	
Age 43	173 (40%)	140 (39%)	33 (45%)	
Age 53	143 (33%)	115 (32%)	28 (38%)	
Age 63	220 (51%)	179 (50%)	41 (55%)	
Age 70	199 (47%)	157 (45%)	42 (58%)	

Participant characteristics within the Insight 46 cohort.

^a^Of whom 51 were identified as night workers (as opposed to night or shift workers). Wilcoxon rank-sum test^b^ and Welch’s two-sample *t*-test^c^ were used to examine differences between night/shift workers and non-shift workers for non-normally and normally distributed continuous outcomes, respectively; Fisher’s exact test was used for all categorical outcomes.

Mortality and dementia diagnosis data were available for 3040 participants from the wider NSHD with night/shift working and covariate data. A total of 1067 NSHD participants had shift work data and plasma p-tau217 available. Participant characteristics in the NSHD cohort and a more detailed breakdown of occupations among night/shift workers and non-shift workers are provided in Supplementary Tables 1–3.

### Associations between night/shift working at age 31 and life course sleep measures

Accounting for sex, socioeconomic position, childhood education and age at actigraphy for analyses including actigraphic measures, we found no significant difference between night/shift workers and non-shift workers in self-rated sleep quality at age 31, 36, 43 or 69 or with TST, SE, WASO, IS or IV measured with actigraphy at age 73 (Supplementary Fig. 1).

### Associations between night/shift working at age 31 and whole-brain, hippocampal and white matter hyper-intensity volume at age 70

**Figure 2:**
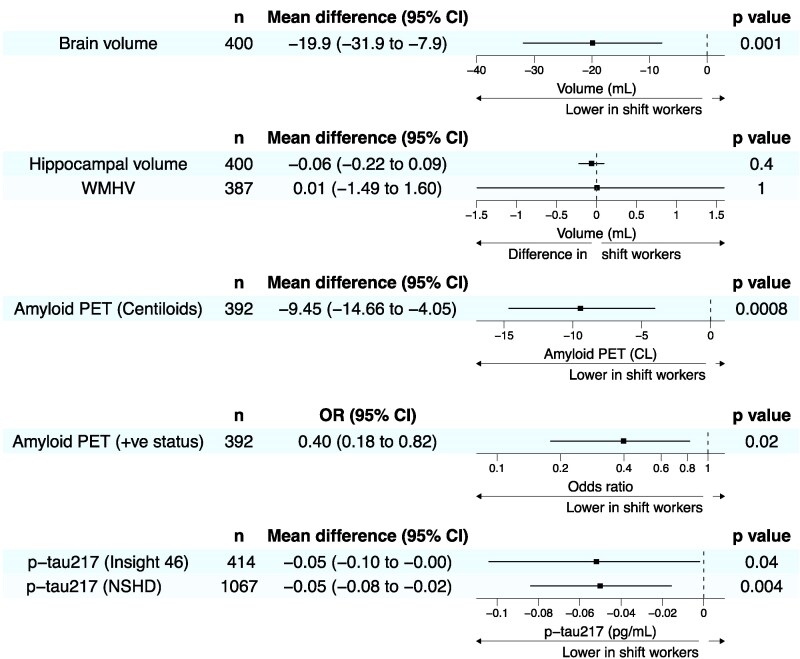
**Night and shift working is associated with lower brain volume and less Alzheimer’s disease pathology.** Forest plots demonstrating lower brain volume, Amyloid PET CLs, amyloid PET positivity and plasma p-tau217 in night/shift workers, without significant difference in hippocampal volume or WMHV by age 70. Plasma p-tau217^‡^ from participants attending Insight 46 with night/shift working and covariate data available (*n* = 414) was supplemented by samples taken from the wider NSHD cohort (*n* = 653) during the same collection period. Linear/logistic regression was used for continuous/binary outcomes, respectively. The mean difference represents the covariate-adjusted difference in the mean imaging or plasma outcomes in night/shift workers, compared with non-shift workers. Case bootstrapping was used for the relationships with WMHV, Amyloid PET CLs and p-tau217, which are not normally distributed. All analyses are adjusted for sex, adult socioeconomic position, educational attainment, age at imaging/plasma, alongside TIV for volumetric measures and *APOE* ɛ4 carrier status for Amyloid PET and plasma p-tau217. *n*, number of participants included in the analysis, having all required data available; CL, Centiloids.

Accounting for sex, socioeconomic position, childhood education, TIV and age at imaging, night/shift workers had significantly smaller whole-brain volumes than non-shift workers (−19.9 mL, 95% CI −31.9, −7.9, *P =* 0.001; Fig. 2).

There was no evidence of a difference in hippocampal or WMHV in night/shift workers (Fig. 2).

Sensitivity analyses demonstrated the relationship with lower brain volume was weaker when examined in night workers (*n* = 51) (rather than night *or* shift workers, *n* = 74) (−14.3 mL, 95% CI −29.0, 0.3; *P =* 0.05) but remained similar when adjusting for *APOE* ɛ4 carrier status (−21.2 mL, 95% CI −33.2, −9.3; *P =* 0.0005), Aβ PET Centiloids (−20.0 mL, 95% CI −32.3, −7.6; *P =* 0.002), childhood cognition (−19.9 mL, 95% CI −31.9, −7.8; *P =* 0.001) and after excluding those (*n* = 40, Supplementary Table 4) with significant brain disorders from the analysis (−18.1 mL, 95% CI −30.4, −5.8, *P =* 0.004). There was no significant interaction with sex in the relationship between shift working and brain volume (*P* = 0.3).

### Associations between night/shift working at age 31 and Alzheimer’s disease pathology at age 70

Accounting for sex, socioeconomic position, childhood education, *APOE* ɛ4 carrier status and age at imaging, night/shift workers had significantly lower Aβ PET (−9.45 Centiloids, 95% CI −14.7, −4.1, *P =* 0.0008, Fig. 2) and lower rates of positive Aβ PET status [odds ratio (OR) 0.40, 95% CI 0.18, 0.82, *P =* 0.02] than non-shift workers. Night/shift workers also had lower plasma p-tau217 concentrations both when this was examined in the Insight 46 sample (−0.05 pg/mL, 95% CI −0.10, −0.001, *P =* 0.04) and in the wider NSHD cohort (*n* = 1067 with available data, Supplementary Table 1) (−0.05 pg/mL, 95% CI −0.08, −0.02, *P =* 0.004).

The relationship with lower Aβ PET Centiloids remained when examined specifically in night workers (rather than night *or* shift workers) (−9.8 Centiloids, 95% CI −15.8, −3.6; *P =* 0.003), after adjusting for childhood cognition (−9.3 Centiloids, 95% CI −14.5, −3.9; *P =* 0.001), without adjustment for *APOE* ɛ4 carrier status (−8.0 Centiloids, 95% CI −13.4, −2.4; *P =* 0.006), and after excluding those with significant brain disorders from the analysis (−8.8 Centiloids, 95% CI −14.2, −3.1, *P =* 0.003).

As expected, *APOE* ɛ4 allele carriers had significantly higher Aβ PET burden (19.1 Centiloids, 95% CI 13.5, 24.9; *P <* 0.0001), higher rates of positive Aβ PET status (OR 5.4, 95% CI 3.4, 8.6, *P* < 0.0001) and higher plasma p-tau217 concentration (0.12, 95% CI 0.09, 0.16, *P* < 0.0001) than non-carriers. There was no significant interaction with sex in the relationship between shift working and Aβ PET Centiloids (*P* = 0.7), Aβ PET status (*P* = 0.8) or plasma p-tau217 concentration (*P* = 0.9).

These results did not appear to be driven by mortality since there was no significant difference in mortality between night/shift workers and non-shift workers in the NSHD cohort by age 70 [hazard ratio (HR) 1.02, 95% CI 0.78, 1.33, *P* = 0.9, *n* = 3039 with night/shift working, mortality, and covariate data].

### Associations between night/shift working and late-life cognition

Accounting for sex, socioeconomic position, childhood education, childhood cognition and age at cognitive assessment, there was no significant relationship between night/shift working and late-life cognition assessed using the PACC at age 70 (0.09, 95% CI −0.04, 0.2, *P* = 0.2).

### Associations between night/shift working and dementia diagnosis

By age 78, of 3040 NSHD participants with available data, 4 (0.9%) night/shift workers and 72 (2.8%) non-shift workers had been diagnosed with all-cause (excluding vascular) dementia and 3 (0.7%) of night/shift workers and 12 (0.5%) of non-shift workers had been diagnosed with vascular dementia (Supplementary Table 2).

**Figure 3:**
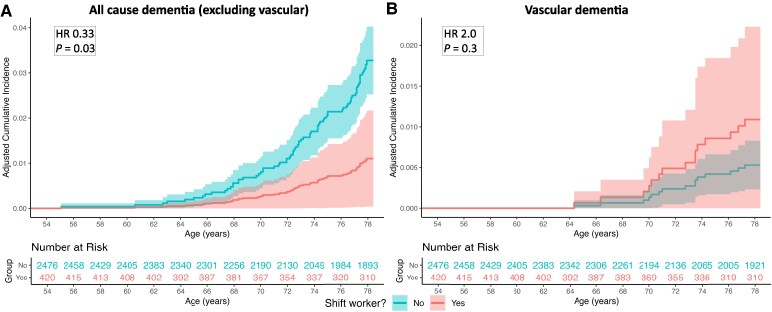
**Night/shift working is associated with risk of dementia.** Covariate-adjusted cumulative incidence plots demonstrating incidence of all-cause (excluding vascular) dementia (**A**) and vascular dementia (**B**) in night/shift workers compared with non-shift workers. Cox proportional cause-specific hazards regression models were used to compare rates of dementia diagnosis in night/shift workers and non-shift workers, censoring for the competing event of death and for age at emigration by age 78.0 years, and controlling for sex, educational attainment and socioeconomic position. *n* = 3040 for both analyses. Shaded areas represent the 95% CI. Plots are truncated to show only years during which all-cause dementia diagnoses occurred.

Accounting for sex, socioeconomic position and childhood education, night/shift workers had significantly lower rates of all-cause (excluding vascular) dementia (HR 0.33, 95% CI 0.12, 0.92, *P* = 0.03, Fig. 3A), compared to non-shift workers. Night shift workers had an increased rate of vascular dementia (HR 2.0, 95% CI 0.5, 8.1, *P* = 0.3, Fig. 3B), although noting very small numbers, this was not statistically significant.

Sensitivity analyses demonstrated that the significant relationship between night/shift working and reduced all-cause (excluding vascular) dementia risk remained similar after additionally controlling for *APOE* ɛ4 allele carriage (HR 0.27, 95% CI 0.08, 0.89, *P* = 0.03) or childhood cognition (HR 0.36, 95% CI 0.13, 0.99, *P* = 0.048). There was no significant interaction with sex in the relationship between shift working and dementia risk (*P* = 1).

*APOE* ɛ4 allele carriers had significantly higher rate of all-cause (excluding vascular) dementia diagnosis compared to non-carriers (HR 1.9, 95% CI 1.2, 3.0, *P* = 0.009).

### Associations between night/shift working and life course health and behaviours

**Figure 4:**
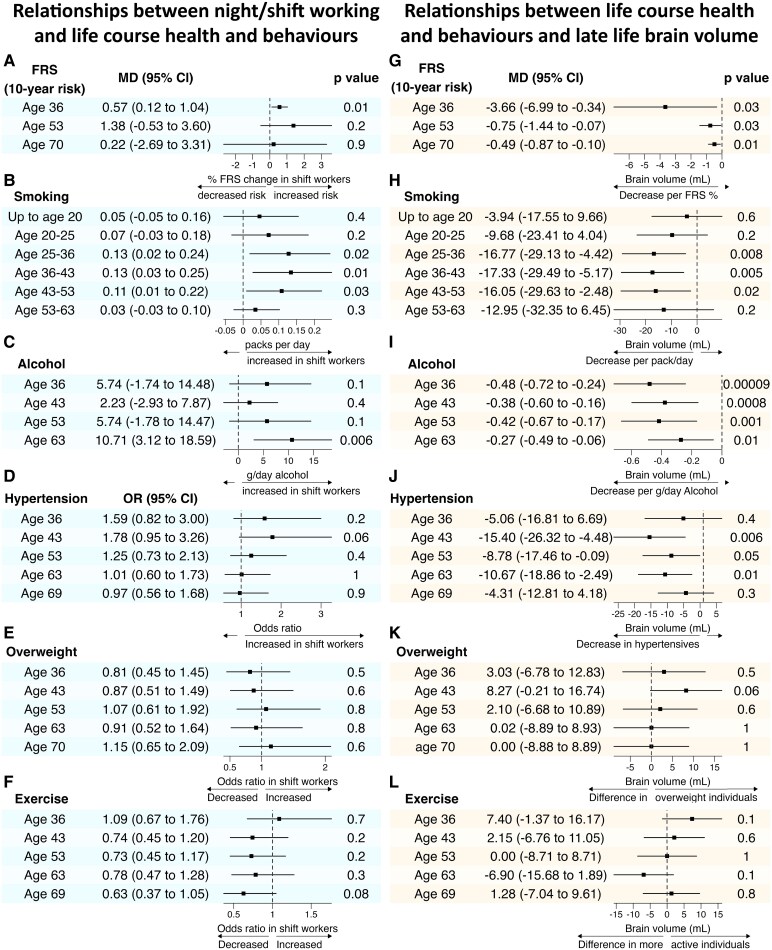
**Night/shift working is related to life course health and behaviours, and life course risk factors are related to reduced brain volume.** Forest plots demonstrating associations between night/shift working and cardiovascular risk factors/alcohol use across the life course (**A–F**) and the relationship between these factors and brain volume in the eighth decade (**G–L**). Linear/logistic regression was used for continuous/binary outcomes, respectively. Case bootstrapping was used where FRS, alcohol consumption or smoking are outcomes in the regression model as these are not normally distributed. Smoking is presented as the mean packs smoked per day during the presented time interval, or averaged over 5 years for those smoking prior to the age of 20. Sex, adult socioeconomic position and educational attainment were used as covariates in all analyses, alongside TIV and age at imaging for analyses including brain volume. Sample size varied for each analysis due to data availability: (**A**) FHS age 36 (*n* = 408), age 53 (*n* = 418) and age 70 (*n* = 428); (**B**) smoking up to age 20 (*n* = 408), age 20–25 (*n* = 407), age 25–36 (*n* = 403), 36–43 (*n* = 411), 43–53 (*n* = 416) and 53–63 (*n* = 403); (**C**) alcohol age 36 (*n* = 347), age 43 (*n* = 418), age 53 (*n* = 318) and age 63 (*n* = 309); (**D**) hypertension age 36 (*n* = 412), age 43 (*n* = 417), age 53 (*n* = 411), age 63 (*n* = 430) and age 69 (*n =* 423); (**E**) overweight age 36 (*n* = 412), age 43 (*n* = 419), age 53 (*n* = 423), age 63 (*n* = 431) and age 70 (*n* = 431); (**F**) exercise age 36, 43, 54 and 63 (*n* = 431), age 69 (*n* = 424); (**G**) FRS age 36 (*n* = 423), age 53 (*n* = 454) and age 70 (*n* = 463); (**H**) smoking up to age 20 (*n* = 427), age 20–25 (*n* = 421), age 25–36 (*n* = 391), age 36–43 (*n* = 430), age 43–53 (*n* = 437) and age 53–63 (*n* = 419); (**I**) alcohol age 36 (*n* = 360), alcohol age 43 (*n* = 445), alcohol age 53 (*n* = 340) and alcohol age 63 (*n* = 341); (**J**) hypertension age 36 (*n* = 426), age 43 (*n* = 445), age 53 (*n* = 441), age 63 (*n* = 467) and age 69 (*n* = 456); (**K**) overweight age 36 (*n* = 429), age 43 (*n* = 449), age 53 (*n* = 459) and age 63 and 70 (*n* = 468); (**L**) exercise age 36, 43, 53 and 63 (*n* = 468) and age 69 (*n* = 461). FRS, Framingham risk score; MD, adjusted mean difference.

Night/shift workers had a 0.6% higher FRS (95% CI 0.12, 1.0; *P =* 0.01, Fig. 4A) at age 36 and a trend towards higher FRS at age 53. Night/shift workers smoked more at all timepoints measured from age 25–53 (Fig. 4B), smoking an additional 5.9 pack-years by age 53 (95% CI 1.7, 10.4; *P =* 0.005), equivalent to 42 975 additional cigarettes per person.

There was a trend towards higher alcohol consumption among night/shift workers across the life course, reaching statistical significance at age 63, when they consumed an average 10.7 g/day more alcohol (95% CI 3.1, 18.6; *P =* 0.006, Fig. 4C) than non-shift workers, equivalent to an additional ∼10 L of spirits annually.

Non-significant trends were seen towards increased rates of hypertension at ages 36 and 43 in night/shift workers (Fig. 4D) and less exercise from age 43 onwards (Fig. 4F). There was no significant difference in the proportion becoming overweight (Fig. 4E) at any timepoint.

### Selection of potential mediators for effect of life course health and behaviours on smaller brain volume

Higher FRS and increased alcohol consumption were significantly associated with lower late-life brain volume at all timepoints measured, as was smoking from age 25–53 and hypertension age 43–63, with a trend towards lower brain volume in individuals with hypertension at all other timepoints. There was no significant relationship between brain volume and exercise or being overweight (Fig. 4G–L). Therefore alcohol, smoking and hypertension were chosen as candidate mediators.

### Selection of potential mediators for effect of life course health and behaviours on amyloid-β PET

There was no significant relationship between FRS, smoking, alcohol consumption, hypertension, exercise or being overweight at any measured timepoint and Aβ PET Centiloids at age 70. There was therefore no evidence of a significant mediation effect of these factors in the relationship between night/shift working and Aβ PET Centiloids. Therefore, no mediation analysis was conducted for this outcome.

### Causal mediation analysis

**Figure 5:**
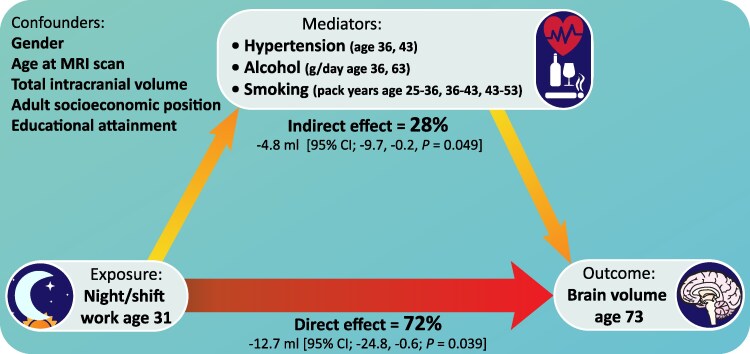
**Reduced brain volume in shift workers is partially mediated by increased life course cardiovascular risk factors.** Causal mediation analysis examined the extent to which the relationship between night/shift working and brain volume was mediated by life course factors. Using a natural-effects model approach, this model included those life course risk factors shown to be most strongly related to both shift working and brain volume (defined as *P* ≤ 0.15 within 5 years of night/shift working, *P* ≤ 0.1 at later timepoints, see Fig. 4). FRS was represented by its constituent components (smoking, blood pressure and anti-hypertensive medications use) except for diabetes status, which was not included due to there being only one individual with diabetes at age 36. Mediation analyses were performed on multiply imputed datasets to mitigate potential bias from missing data, using multiple imputation of covariates by SMC-FCS (150 iterations, 150 imputations), including all participants with brain volume data available (*n* = 468).

Causal mediation analysis examined the extent to which lower brain volumes in night/shift workers at age 70 were mediated by hypertension, alcohol and smoking (Fig. 5).

Out of the total effect of night/shift working on brain volume [−17.6 mL (95% CI −29.0, −6.1), *P* = 0.003], the natural direct effect was −12.7 mL (95% CI −24.8, −0.6; *P =* 0.039). The natural indirect effect of shift working on brain volume mediated by hypertension (ages 36 and 43), alcohol consumption (ages 36 and 63) and smoking pack-years (ages 25–36, 36–43 and 43–53) was −4.8 mL (95% CI −9.7, −0.2, *P =* 0.049). Thus 28% of the observed effect of night/shift working on reduced brain volume is mediated by these measured cardiovascular risk factors and alcohol consumption (Fig. 5). This total effect differs slightly from that reported in Fig. 2 due to the use of multiple imputation to maximize sample size (*n* = 468).

## Discussion

We demonstrate that night/shift working in the fourth decade is associated with significant effects on brain health >40 years later. Night/shift workers had lower brain volume and lower levels of Alzheimer’s disease pathology in late-life, without any significant relationship with hippocampal volume or WMHV. By age 78, dementia diagnoses in night/shift workers had occurred at one-third of the rate seen in the non-shift workers.

The lower brain volume among night/shift workers is equivalent to 3.4 years additional aging as assessed in previous longitudinal analysis of the cohort.^54^ Reduced brain volume predicts cognitive decline in elderly individuals^55^ and would be anticipated to be associated with increase dementia risk. The lower rates of dementia among night/shift workers reported here are most likely due to a greater influence of the unexpected finding of reduced levels of Alzheimer’s disease pathology.

Although not significant (noting very small numbers), there was a suggestion that night/shift workers may have increased rates of vascular dementia, which would be unsurprising given their increased exposure to cardiovascular risk factors throughout the life course. We found no difference in cognition between shift and non-shift workers at age 70; however, this may have been impacted by relatively low rates of dementia at this age and a reluctance to attend a 2-day study visit among those with incipient cognitive decline.

Night/shift workers did not report increased rates of sleep disturbance at any measured timepoint, while their sleep in late-life was no different to non-shift workers as measured objectively with actigraphy. This suggests that it may be circadian disruption, rather than disturbed sleep, that accounts for the findings presented here. The subjective assessment of sleep quality at early timepoints was relatively limited however, while multiple studies report impairment of objective and subjective measures of sleep quality among shift workers,^56,57^ noting an over-representation of healthcare professionals in the literature and that some studies report increased TST with some shift patterns.^57-60^

Despite having less healthy lifestyles, higher rates of *APOE* ɛ4 allele carriage and smaller brains, night/shift workers had lower levels of Alzheimer’s disease pathology as measured by both amyloid PET and plasma p-tau217. This was unexpected, given the growing literature suggesting a causative role of sleep and circadian disruption in Alzheimer’s disease via amyloidogenic pathways. However, many of those studies suggesting an amyloidogenic effect of sleep disturbance utilize acute sleep deprivation paradigms quite different to the chronic circadian disruption explored here,^3,5,61^ and few if any explore the influence of sleep disturbance this early in the life course.

Previous studies suggesting that shift workers are at increased risk of all-cause dementia predominantly examine shift working in later life, are unable to account for important occupation and dementia associated life course factors such as socioeconomic position and education and generally have a relatively short follow-up of <15 years meaning that prodromal Alzheimer’s disease pathology is likely to already be present in many of those who go on to develop dementia and may have impacted employment.^17,19^ It has also been shown that deleterious cognitive effects of shift work recover following cessation of shift working for at least five years.^62^ Such a recovery may well be relevant to many of the shift workers examined herein, given that we only observe shift working at a single timepoint relatively early in life, and many are likely to have changed their subsequent occupation or working patterns. It is also possible that differential relationships between Aβ accumulation and later-life shift working are present, perhaps due to failure of compensatory mechanisms or increased susceptibility of the aging brain.^63^

Possible explanations for the unexpectedly reduced rate of Alzheimer’s disease pathology in night/shift workers include selection bias, although this is unlikely to explain night/shift workers having smaller brains and numerous risk factors for Alzheimer’s disease but less amyloid pathology, while mortality by age 70 was unrelated to night/shift working. The result is further corroborated by reduced dementia rates described herein, derived from robustly collected national statistics. Alternatively, those with more resilient brains may be more tolerant of the physiological effects of night/shift work and therefore more likely to undertake it. Finally, this result raises the prospect that night/shift working could confer a benefit on brain health, at least when undertaken relatively early in life. Given that glymphatic clearance of pathological proteins from the brain appears most effective during slow-wave sleep,^5^ deteriorates with age^63^ and may be enhanced by factors including exercise or sensory stimulation,^64,65^ one possibility is that night/shift working in early life may enhance the robustness of this system in late-life, the equivalent of attending the ‘glymphatic gym’. Such an adaptation would be analogous to cardiac remodelling in response to physical exercise,^66^ or synaptic plasticity following injury or stimulation of the CNS.^67^

Understanding the mechanism for this finding will require further study, while recent evidence that brain clearance of some molecules is reduced during sleep has similarly questioned the glymphatic hypothesis linking sleep and Alzheimer’s disease.^68^

Surprisingly, we found no relationship between night/shift working and WMHV. Given that cardiovascular risk has a ‘dose-dependent’ relationship with WMHV,^30,33^ the lack of such a relationship here may reflect reduced power, noting that only 17% of participants were night/shift workers. Alternatively, this may be related to those with significant vascular disease being less likely to attend for a study visit, given that we did find a suggestion of increased rates of vascular dementia diagnoses among night/shift workers in the wider NSHD cohort. The lack of association between life course cardiovascular risk and amyloid pathology has been demonstrated previously in this cohort and likely reflects the contribution of different pathologies to dementia risk.^30,33^

## Strengths and limitations

Strengths of this study include the size, very small age range of the cohort, >40-year follow-up and extensive prospectively collected data enabling robust correction for life course determinants of brain health. Health episode statistics provide a robust reporting mechanism for dementia diagnoses, although it is well recognized that clinical diagnosis correlates relatively poorly with underlying pathological dementia diagnosis, with high rates of mixed pathology,^43,69^ necessitating grouping together of presumed neurodegenerative and all-cause dementia diagnoses herein. Age 78 remains a relatively young age for dementia diagnosis; longer term follow-up is ongoing in this cohort, including tau PET imaging.

Despite night/shift workers and non-shift workers being well matched with regards to education, childhood cognition and socioeconomic position, it remains possible that unmeasured confounders could influence both the participants’ selection of jobs entailing shift work and their life course cardiovascular risk or brain health. With a single exception, NSHD participants are White, reflecting the British post-war population from which it was formed, potentially limiting applicability of these results across different populations. Those completing Insight 46 had higher educational attainment, socioeconomic position and cognition and were healthier than the general NSHD population.^27^ Underrepresentation of those with life course cardiovascular risk factors may reduce the effect sizes related to these factors. We were unable to examine the type or duration of night/shift working, noting that the latter is likely a significant determinant of effect size.^18^

## Conclusion

Despite having less healthy lifestyles, higher rates of *APOE* ɛ4 allele carriage and smaller brains, night/shift workers had lower levels of Alzheimer’s disease pathology and lower rates of all-cause (excluding vascular) dementia. While the lower brain volume in night/shift workers, equivalent to 3.4 years additional aging, is partially mediated by life course cardiovascular risk factors, the majority of the effect is independent of these measured modifiable lifestyle behaviours and may be a consequence of chronic sleep/circadian disruption or related to other unmeasured factors. Lower levels of Alzheimer’s disease pathology and dementia among night/shift workers were unexpected; further study is required to understand the mechanism.^70^

## Supplementary Material

fcaf264_Supplementary_Data

## Data Availability

The NSHD data-sharing policy is available on the NSHD Data Sharing website (https://nshd.mrc.ac.uk/data-sharing). Variables used for analysis are collated in basket joshkrZZgkoomk on the Condor data-sharing platform.
