# Supplementary material for: Associations between night/shift working and late-life brain health
Source: Brain Commun. 2025 Jul 4;7(4):fcaf264. doi: 10.1093/braincomms/fcaf264 (PMC12268499; doi:10.1093/braincomms/fcaf264)
Supplement: fcaf264_Supplementary_Data [file fcaf264_supplementary_data.pdf]

Supplementary Materials

	n	Estimate (95% CI)	p value
	**n**	**Odds ratio (95% CI)**	**p value**
Trouble with sleep? (age 31)	3014	1.14 (0.89 to 1.44)	0.3
	**n**	**Mean difference (95% CI)**	**p value**
Trouble initiating sleep? (age 36)	2696	-0.01 (-0.04 to 0.01)	0.3
	**n**	**Mean difference (95% CI)**	**p value**
Trouble maintaining sleep? (age 36)	2704	0.00 (-0.02 to 0.02)	0.7
	**n**	**Mean difference (95% CI)**	**p value**
Trouble initiating sleep? (age 43)	2565	0.05 (-0.08 to 0.18)	0.5
	**n**	**Mean difference (95% CI)**	**p value**
Trouble maintaining sleep? (age 43)	2568	0.04 (-0.10 to 0.19)	0.6
	**n**	**Mean difference (95% CI)**	**p value**
Total sleep time (age 73)	374	-0.03 (-0.27 to 0.20)	0.8
	**n**	**Mean difference (95% CI)**	**p value**
Sleep efficiency (age 73)	374	-0.94 (-2.37 to 0.43)	0.2
	**n**	**Mean difference (95% CI)**	**p value**
WASO (age 73)	374	3.87 (-0.77 to 8.75)	0.1
	**n**	**Mean difference (95% CI)**	**p value**
Interdaily stability (age 73)	374	0.01 (-0.02 to 0.04)	0.6
	**n**	**Mean difference (95% CI)**	**p value**
Intradaily variability (age 73)	374	0.04 (-0.02 to 0.10)	0.2

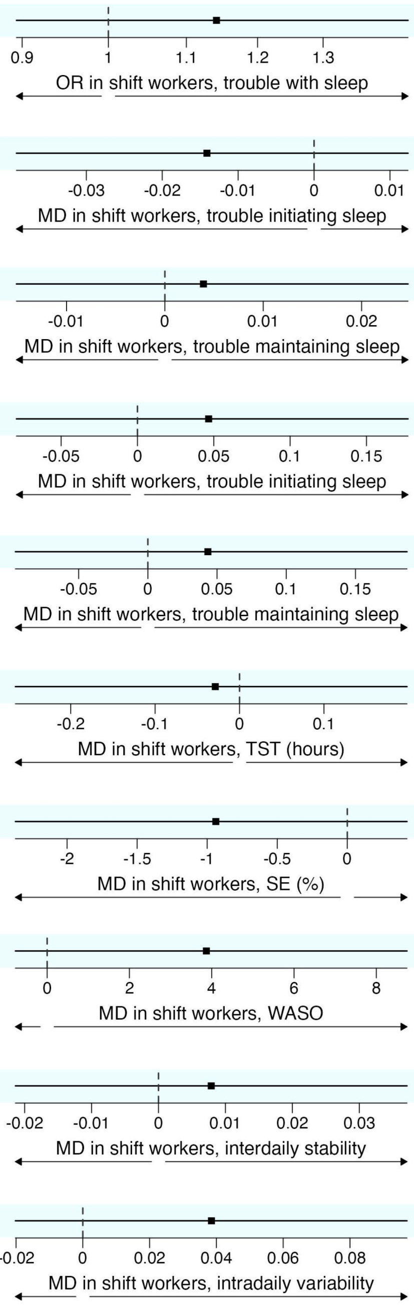

Supplementary Figure 1: Associations between night/shift working and measures of sleep and circadian function throughout the life course. Forest plots demonstrating associations between night/shift-working, self-reported sleep quality across the life course, and sleep and circadian function assessed with actigraphy at age 73. Linear regression with case bootstrapping and logistic regression were used for continuous and binary outcomes, respectively. Results are presented as the mean difference (MD) in listed actigraphy metric or sleep disturbance score (higher representing more disturbed sleep for self-reported measures) among night/shift workers compared to non-shift-workers for subjective measures of sleep quality, with the exception of binary outcome measures, where we present the odds ratio (OR) of reporting sleep disturbance among night/shift-workers. WASO; wake after sleep onset (minutes), TST; total sleep time (hours), SE; sleep efficiency (%).

Supplementary Table 1: Participant characteristics, NSHD plasma p-tau217 sample

Characteristics	**Overall**, N = 1067	Night/shift-worker? **No**, N = 897	Night/shift-worker? **Yes**, N = 170	**p-value**
Age at plasma sampling, years, mean (SD)	69.9 (0.7)	69.9 (0.7)	69.9 (0.7)	0.4
Female sex, n (%)	547 (51%)	507 (57%)	40 (24%)	<0.001
Adult socioeconomic position, n (%)				0.03
Professional	84 (7.9%)	68 (7.6%)	16 (9.4%)	
Intermediate	467 (44%)	399 (44%)	68 (40%)	
Skilled (non-manual)	254 (24%)	222 (25%)	32 (19%)	
Skilled (manual)	138 (13%)	104 (12%)	34 (20%)	
Partly/unskilled	124 (12%)	104 (12%)	20 (12%)	
Highest educational qualification, n (%)				0.4
None	270 (25%)	226 (25%)	44 (26%)	
Below O-levels (vocational)	81 (7.6%)	73 (8.1%)	8 (4.7%)	
O-levels or equivalent (secondary school)	243 (23%)	202 (23%)	41 (24%)	
A-levels or equivalent (higher secondary/college)	334 (31%)	284 (32%)	50 (29%)	
Degree or equivalent	139 (13%)	112 (12%)	27 (16%)	
Childhood cognition, z-score, mean (SD)	0.19 (0.79)	0.21 (0.79)	0.12 (0.79)	0.2
APOE ε4 carrier, n (%)	330 (31%)	265 (30%)	65 (38%)	0.025
PSQI, global score age 69, mean (SD)	4.9 (3.2)	4.9 (3.2)	4.7 (3.1)	0.7

Participant characteristics within the NSHD sample. Including only those with plasma p-tau217, night/shift-work, and covariate data available. This sample includes those (n = 416) with plasma p-tau217 from Insight 46. SD; standard deviation, WMHV; white matter hyperintensity volume, PSQI; Pittsburgh Sleep Quality Index. Wilcoxon rank-sum test was used to examine differences between night/shift-workers and non-shift-workers for continuous outcomes, Fisher's exact test was used for all categorical outcomes.

Supplementary Table 2: Participant characteristics, NSHD dementia survival analysis sample

Characteristics	**Overall**, N = 3,040	Night/shift-worker? **No**, N = 2,597	**Yes**, N = 443	p-value
Female sex, n (%)	1,523 (50%)	1,427 (55%)	96 (22%)	<0.001
Adult socioeconomic position, n (%)				<0.001
Professional	202 (6.6%)	161 (6.2%)	41 (9.3%)	
Intermediate	1,039 (34%)	897 (35%)	142 (32%)	
Skilled (non-manual)	722 (24%)	656 (25%)	66 (15%)	
Skilled (manual)	575 (19%)	445 (17%)	130 (29%)	
Partly/unskilled	502 (17%)	438 (17%)	64 (14%)	
Highest educational qualification, n (%)				0.7
None	1,143 (38%)	969 (37%)	174 (39%)	
Below O-levels (vocational)	235 (7.7%)	208 (8.0%)	27 (6.1%)	
O-levels or equivalent (secondary school)	642 (21%)	549 (21%)	93 (21%)	
A-levels or equivalent (higher secondary/college)	735 (24%)	682 (24%)	107 (24%)	
Degree or equivalent	285 (9.4%)	243 (9.4%)	42 (9.5%)	
Childhood cognition, z-score, mean (SD)	0.03 (0.83)	0.04 (0.84)	-0.01 (0.80)	0.2
APOE ε4 carrier, n (%)	645 (31%)	541 (31%)	104 (34%)	0.3
PSQI, global score age 69, mean (SD)	5.0 (3.2)	5.0 (3.2)	4.7 (3.0)	
Dementia diagnosis				
All cause (excluding vascular), n (%)	76 (2.5%)	72 (2.8%)	4 (0.9%)	
Vascular dementia, n (%)	15 (0.5%)	12 (0.5%)	3 (0.7%)	

Participant characteristics within the NSHD sample. Including only those with plasma p-tau217, night/shift-work, and covariate data available. This sample includes those (n = 416) with plasma p-tau217 from Insight 46. SD; standard deviation, WMHV; white matter hyperintensity volume, PSQI; Pittsburgh Sleep Quality Index. Wilcoxon rank-sum test was used to examine differences between night/shift-workers and non-shift-workers for continuous outcomes, Fisher's exact test was used for all categorical outcomes.

Supplementary Table 3: Detailed breakdown of occupations undertaken by night/ shift workers and non shift-workers in the NSHD plasma p-tau217 sample.

Occupation	Night/shift worker?	
	**Yes**, N = 170	**No**, N = 897
Production and manufacturing	25 (15%)	50 (5.6%)
Construction and skilled trades	17 (10%)	54 (6.0%)
Management and senior administration	16 (9.4%)	110 (12%)
Nursing	15 (8.8%)	16 (1.8%)
Technical and engineering	14 (8.2%)	81 (9.0%)
Transport and logistics	14 (8.2%)	21 (2.3%)
Emergency services and related occupations	14 (8.2%)	1 (0.1%)
Armed forces	11 (6.5%)	3 (0.3%)
Art, media, sport	9 (5.3%)	11 (1.2%)
Health and social care (excluding nursing)	7 (4.1%)	26 (2.9%)
Education	6 (3.5%)	80 (8.9%)
Clerical and secretarial	5 (2.9%)	136 (15%)
Sales and service	4 (2.4%)	79 (8.8%)
Cleaners and related occupations	4 (2.4%)	19 (2.1%)
Science and research	4 (2.4%)	13 (1.4%)
Professional and finance	2 (1.2%)	24 (2.7%)
Agriculture and natural resources	2 (1.2%)	19 (2.1%)
Inadequately described occupations	1 (0.6%)	11 (1.2%)
Not formally employed	0 (0%)	143 (16%)

Occupations were classified according to Office of Population Censuses and Surveys (OPCS) groupings, which includes 223 different occupational groups, of which only 20 are undertaken by >1% of the cohort. These are further grouped above according to sector due to the large number of occupations undertaken by very small numbers, particularly among production, manufacturing, construction and skilled trade occupations.

Supplementary Table 4: Significant brain disorders

Brain disorder	Frequency
Stroke	17
Epilepsy	5
Multiple Sclerosis	3
Bipolar disorder requiring anti-psychotic medication	2
Alzheimer's Disease	2
Parkinson's disease	2
Depression requiring electroconvulsive therapy	2
Brain metastasis	1
Subdural haematomas requiring neurosurgery	1
Myotonic dystrophy	1
Parkinson's disease and epilepsy	1
Traumatic brain injury	1
Hepatic encephalopathy	1
Dementia (not otherwise specified)	1

Significant brain disorders were identified from the medical history collected during Insight 46 study visit, following review of MRI brain imaging review by a consultant neuroradiologist, or if clear evidence of significant cognitive impairment (MMSE score $\leqslant$ 24 and/or significant concern from study clinician) or clinically detectable parkinsonism (fulfilling Queen Square Brain Bank criteria) was identified during the study visit.
